# Multi-Target Cinnamic Acids for Oxidative Stress and Inflammation: Design, Synthesis, Biological Evaluation and Modeling Studies

**DOI:** 10.3390/molecules24010012

**Published:** 2018-12-20

**Authors:** Eleni Pontiki, Dimitra Hadjipavlou-Litina

**Affiliations:** Department of Pharmaceutical Chemistry, School of Pharmacy, Faculty of Health Sciences, Aristotle University of Thessaloniki, 54124 Thessaloniki, Greece

**Keywords:** cinnamic acids, multitarget, antioxidant, lipoxygenase inhibition, lipid peroxidation

## Abstract

Inflammation is a complex phenomenon that results as a healing response of organisms to different factors, exerting immune signaling, excessive free radical activity and tissue destruction. Lipoxygenases and their metabolites e.g., LTB_4_, are associated with allergy, cell differentiation and carcinogenesis. Lipoxygenase 12/15 has been characterized as a mucosal-specific inhibitor of IgA and a contributor to the development of allergic sensitization and airway inflammation. Development of drugs that interfere with the formation or effects of these metabolites would be important for the treatment of various diseases like asthma, psoriasis, ulcerative colitis, rheumatoid arthritis, atherosclerosis, cancer and blood vessel disorders. In this study we extended our previous research synthesizing a series of multi-target cinnamic acids from the corresponding aldehydes with suitable 4-OH/Br substituted phenyl acetic acid by Knoevenagel condensation. The final products **1i**, **3i**, **3ii**, **4i**, **6i**, **6ii**, and **7i** were obtained in high yields (52–98%) Their structures were verified spectrometrically, while their experimentally lipophilicity was determined as R_M_ values. The novel derivatives were evaluated for their antioxidant activity using DPPH, hydroxyl radical, superoxide anion and ABTS^+•^, anti-lipid peroxidation and soybean lipoxygenase inhibition assays. The compounds presented medium interaction with DPPH (30–48% at 100 µM). In contrast all the synthesized derivatives strongly scavenge OH radicals (72–100% at 100 µM), ABTS^+•^ (24–83% at 100 µM) and presented remarkable inhibition (87–100% at 100 µM) in linoleic acid peroxidation (AAPH). The topological polar surface of the compounds seems to govern the superoxide anion scavenging activity. Molecular docking studies were carried out on cinnamic acid derivative **3i** and found to be in accordance with experimental biological results. All acids presented interesting lipoxygenase inhibition (IC_50_ = 7.4–100 µM) with compound **3i** being the most potent LOX inhibitor with IC_50_ = 7.4 µM combining antioxidant activities. The antioxidant results support the LOX inhibitory activities. The recorded in vitro results highlight compound **3i** as a lead compound for the design of new potent lipoxygenase inhibitors for the treatment of asthma, psoriasis, ulcerative colitis, rheumatoid arthritis, atherosclerosis, cancer and blood vessel disorders.

## 1. Introduction

The use of antioxidants and anti-inflammatories for the prevention and inhibition of natural phenomena and their use as industrial additives or therapeutic agents has been widely recognised for a long time [1,2]. Moreover, inflammatory processes, especially the lipoxygenase pathway, results in the production of leukotrienes (LTs). LTB_4_ are associated with increased interleukin production [3] transcription [4] and neutrophil-dependent hyperalgesia [5]. These properties imply a significant role for LTB_4_ in the pathogenesis of inflammatory diseases such as allergies, arthritis, psoriasis, inflammatory bowel disease, asthma, cell differentiation and carcinogenesis via a free radical mechanism. Moreover inflammatory bowel disease (IBD) is a complex disorder based to a deregulated immune response with undetermined etiology. Studies have shown that inducible nitric oxide synthase is overexpressed resulting high levels of NO generation [6,7]. Since no therapies are available for this disease there is an extreme need the development of new targets. Toumi and co-workers [8] have studied the effect of a probiotic cocktail as a potential candidate for the treatment of IBD.

Additionally, research studies have revealed the importance of ROS in diseases associated with oxidative stress (e.g., atherosclerosis, inflammatory injury, cancer and cardiovascular diseases) [9,10,11,12,13]. In the above diseases reactive oxygen species (ROS) such as superoxide anion, hydroxyl radical and hydrogen peroxide can attack lipids, proteins and DNA [14] and cause proliferation, genetic instability, chemoresistance, angiogenesis, radioresistance and invasion [15]. During the last decade scientists have been focused on multi-target approaches instead of a single molecule hitting one target, based to the complexity and the detrimental effects of ROS in the various diseases in which inflammatory processes are implicated [16]. Drugs that combine the above activities can be beneficial for the treatment of the abovementioned pathophysiological diseases.

Cinnamic acids and their phenolic derivatives such as caffeic, ferulic and sinapic acids have been considered attractive potential antioxidant and anti-inflammatory agents by many research groups due to the multifunctional activities they present. Furthermore they have been found to present potent anti-inflammatory [17,18,19], antioxidative [10], anticancer [20], cardiovascular [21] and antimicrobial [22] properties. Especially, *p*-coumaric acid or 4-hydroxy-*trans*-cinnamic acid presents antioxidant activity involving direct scavenging of ROS by minimizing the oxidation of low-density lipoprotein (LDL) [23].

Althoug H-Natural and synthetic phenolic antioxidants such as stilbenoids, coumarins, flavonoids and carboxylic acids are part of everyday diet preventing oxidative damage in living organisms they seem to present limitations mainly due to their low lipophilicity [24,25]. To overcome this, synthetic efforts are oriented towards new cinnamic acids combined with fragments from other moieties so as to improve their lipophilicity, and biological membrane penetration to reach the desired targets in living organisms [26,27].

**Figure 1:**
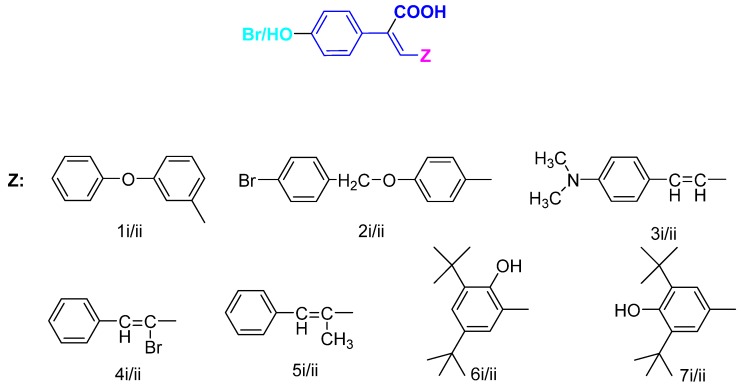
General structure of the novel synthesized derivatives.

Continuing our research on multitarget cinnamic acid derivatives as antioxidant lipoxygenase inhibitors we have designed and synthesized a series of substituted cinnamic acids [17,18,19]. The antioxidant, anti-inflammatory and anticancer properties of cinnamic acids are known to be influenced to a great extent by the substituents on the aryl ring and the double bond [19]. In this study we have tried to modify previously reported phenyl-substituted cinnamic acids with enhanced antioxidant and anti-inflammatory activity in order to improve their physicochemical properties and ameliorate their activity. Thus a series of 4-bromo-/4-hydroxyphenylcinnamic derivatives have been designed and synthesized (Figure 1). In this way we inserted a substituent on the phenyl ring at position 4- and especially a hydroxyl group which can enhance the antioxidant character and a bromine which will influence the overall molar volume and lipophilicity. The new products have been evaluated for hydroxyl radical scavenging activity; superoxide radical scavenging activity; ABTS^+•^ radical cation reduction-decolorization; inhibition of lipid peroxidation and the ability to inhibit soybean lipoxygenase. All novel derivatives are further subjected to modeling studies to compare the in vitro results.

## 2. Results and Discussion

### 2.1. Chemistry

**Scheme 1:**
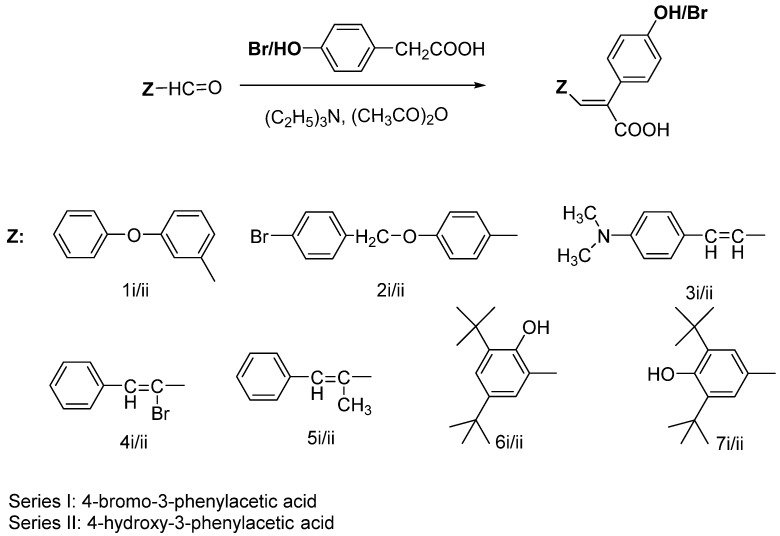
Synthesis of 4-hydroxy-/bromocinnamic acids.

The synthesis of cinnamic acids was achieved by a Knoevenagel condensation as shown in Scheme 1. Acids of series I were derived from the condensation of the suitable cinnamic aldehyde with 4-bromo-3-phenylacetic acid and acetic anhydride in the presence of trimethylamine, while for the acids of series II the same synthetic procedure was used but with 4-hydroxy-3-phenylacetic acid.

The final products **1i**, **3i**, **3ii**, **4i**, **6i**, **6ii**, **7i** were obtained in high (52–98%) and moderate yields (22–43%) (compounds **1ii**, **2i**, **4ii**, **7ii**) with the only exception of compounds **2ii**, **5i** and **5ii** which were obtained in lower yields. The pure final products were recrystallized from ethanol/water. IR, ^1^H-NMR, ^13^C-NMR and elemental analysis were used for the confirmation of the synthesized compounds structures (Table 1). All the acids present the characteristic absorbance in the IR (nujol) (nearly 3200 (O-H), 1720 (C=O), 1625 (C=C), cm^−1^). The ^1^H-NMR and ^13^C-NMR data confirm the proposed structures. According to the spectroscopic data the compounds present *E-*configuration [17,28,29], while characteristic C-NMR-peaks are present e.g., ^1^H-NMR CH=C or e.g., ^13^C-NMR *C*OOH 160–185 ppm. The carboxylic and phenolic protons are not recorded in the ^1^H-NMR spectra when CDCl_3_ [17,28] was used as a solvent. This is commonly happens and referred in the literature. These protons are shown when DMSO [29] is used although this is not always happen due to the presence of water in DMSO.

### 2.2. Physicochemical Studies

Lipophilicity is a physicochemical property correlated with the biological activity. Lipophilicity influences ligand-target binding interactions, solubility, absorption, distribution, bioavailability, metabolism and elimination (ADME) and toxicological outcomes. The lipophilicity of the novel cinnamic acids was experimentally measured as R_M_ values using a reverse phase thin layer chromatography (RPTLC) method [30,31]. This is acknowledged as a reliable, fast and convenient approach for expressing lipophilicity (Table 1). R_M_ relates to the thermodynamic R_f_ value in a logarithmic form. Bate-Smith and Westall firstly determined lipophilicity as R_M_ values in 1950 [31]. The Br-substituted derivatives **1i**–**7i** are more lipophilic than the OH-substituted ones **1ii**–**7ii** which can be easily explained by their structural characteristics.

The in silico determination of lipophilicity—theoretically calculated lipophilicity as clog *P—*was determined using the CLOGP Program of Biobyte Corp. An attempt to correlate log *P* and R_M_ values (Table 1) did not succeed. The different nature of the hydrophilic and lipophilic phases used in the two systems is the main reason.

### 2.3. Biological Evaluation

In this research study, the new substituted cinnamic acids were evaluated for their antioxidant profile using different assays as well as for their ability to inhibit soybean lipoxygenase. Previous publications have proved the antioxidant activity of the several substituted cinnamic acids and their anti-inflammatory activity [17,18,19,32,33]. Different assays were used to assess in vitro antioxidant activity of the novel derivatives involving the generation of different radicals in order to overcome factors such as solubility or steric hindrance which may be of overriding importance in one environment but not in another one. Two different approaches have been considered: (i) the assays measuring the scavenging ability by hydrogen- or electron donation of a preformed free radical as a marker of antioxidant activity as well as (ii) assays involving the presence of an antioxidant system during the generation of the radical. Herein the in vitro antioxidant capacity was monitored in terms of: (a) the interaction with the stable free radical DPPH; (b) the hydroxyl radical scavenging activity; (c) the superoxide radical scavenging activity; (d) the ABTS^+•^ radical cation reduction-decolorization ability and (e) the anti-lipid peroxidation activity. We also investigated the ability of the new acids to inhibit soybean lipoxygenase.

The substituted acids were monitored for the antioxidant activity by the use of the stable 2,2-diphenyl-1-picrylhydrazyl radical (DPPH) at concentration 50 µM and 100 µM after 20 and 60 min (Table 1) [17,18,19,28,33]. In this assay the DPPH radical is reduced by single electron transfer from the antioxidant. Strong antioxidants are the phenoxide anions from phenolic compounds like catechol and derivatives, such as nordihydroguaretic acid (NDGA), the reference compound. The compounds presented medium reducing abilities from 29–61%, due to steric reasons; small differences were found among the compounds with time and concentration. 4-Br-substituted-3 phenyl acetic acid derivatives (Series I) are equipotent to 4-OH-substituted-3 phenyl acetic acid derivatives (Series II). No differences were observed between **4i** and **5i** and **4ii** and **5ii**. On the other hand it seems that the 5-*di*-*tert*-butyl-2-hydroxy derivatives compared to the 5-*di*-*tert*-butyl-4-hydroxy are more potent reducing agents (**6i > 7i** and **6ii > 7ii**).

Competition of the novel substituted acids with DMSO for hydroxyl radicals was measured. Hydroxyl radicals were generated using the Fe^3+^/ascorbic acid system and expressed as a percentage inhibition of formaldehyde production in the presence of each compound at 100 μM (Table 2) [17,18,19,28,33]. All the synthesized derivatives strongly scavenge hydroxyl radicals at 100 μM in comparison to the reference compound Trolox. Compounds **1ii**, **3i**, **6i** and **6ii** are most potent with the exception of **7ii** being slightly less potent than Trolox.

Superoxide radical is a main cause of oxidative stress, expressing its biological toxicity by the inactivation of iron-sulfur cluster containing enzymes [34]. The superoxide anion radical (O_2_^−^^•^) is less toxic than the hydroxyl radical, but still being one of the most known toxic reactive oxygen species (ROS). In this assay the superoxide anion radicals were generated by a non-enzymatically assay and the compounds are tested at 100 μM final concentration (Table 2). Caffeic acid (CA) has been used as a reference compound. Compound **4ii** presented the highest activity, followed by compound **3ii**. We succeeded to derive a quantitative-structure activity relationship model for this activity in which the topological polar surface (TPAS) of the compounds seem to govern the superoxide anion scavenging activity:

% Superoxide Anion Scavenging Activity (100 µM) = 0.019 (±0.007) TPSA + 0.813 (±0.358)

*n* = 6, r = 0.962, r^2^ = 0.926, q^2^ = 0.694, s = 0.122, Q = 7.885, *F_1,6_* = 3.701, α = 0.1 clog *P* vs. TPSA 0.

No role for lipophilicity was found. Oxidation of ABTS with potassium persulfate generates directly the ABTS radical cation (ABTS^+•^) with no involvement of an intermediary radical, which is then reduced upon adding electron-donating antioxidants. Since it is a decolorization assay, the generation of the cation radical does not take place continually in the presence of the antioxidant but the radical is formed prior to the addition of the antioxidant. Most compounds at 100 μΜ showed moderate reducing ability, with the exception of **1ii** (83.4%) and **3i** (83.4%) being as effective as Trolox (Table 2).

We also used water-soluble 2,2′-azinobis(2-amidinopropane) hydrochloride (AAPH) for the generation of free radicals. AAPH has been extensively used as a clean and controllable source of thermally produced alkylperoxyl free radicals [35]. The peroxyl radicals produced by the action of AAPH show total similarity to cellular activities such as lipid peroxidation. All the synthesized derivatives showed remarkable activity at 100 μΜ concentration (84.9–100%) and moderate activity at 10 μΜ (22.6–70.6%) with the exception of **5i** being very weak (9.6%). It seems that the presence of 3,5-*di*-*tert*-butyl-group greatly enhances protection against lipid peroxidation (**6i/ii** and **7i/ii** followed by **3ii** and **5ii**). The lipid peroxidation inhibitory activity seems to be concentration dependent.

The role of lipophilicity (as assessed from calculated clog *P* values, Table 1) is not clear, but the bulk of the substituent seems to play a significant role. Many research groups have used readily obtainable soybean lipoxygenase, which is a homologue of mammalian lipoxygenase and well studied [36,37]. Due to its availability and its well-characterised crystal structure [38], soybean LOX was selected to be used in this study. 4-Br or OH-phenyl substitution does not seem to really affect IC_50_ inhibition (**1i** < **1ii**, **2i** > **2ii**, **5i** > **5ii**, **6i** > **6ii**, **7i** < **7ii**) with the exception of **3** and **4** (Table 2). Compound **3i** presented the highest IC_50_ value of 7.4 µM. Compound **4i** (27.9% inhibition at 100 μΜ) is a very weak inhibitor. Series **5** with a methyl substituent on the conjugated double bond seems to be more potent than the bromo substituent (Series **4**). In general the presence of a conjugated double bond does not seem to affect the activity (Series **3**, **4** and **5**). Bromine is a bulkyy substituent compared to the methyl group and thus a stereochemical effect might influence the inhibition. Although our previous QSAR studies for LOX inhibitors [39] highlight the role of lipophilicity, the present results do not go in parallel to the lipophilicity values. Since LOX inhibitors are acting as radical scavengers, antioxidants, ligands for Fe^3+^ the present results support the correlation of LOX inhibition with the antioxidant activity. From the results it is not well defined if the 4-OH offers to better inhibition since the best molecule **3i** are a 4-Br substituted which points to a hydrophobic and steric interaction from this position.

### 2.4. Computational Studies—Docking Simulation Soybean Lipoxygenase

#### Molecular Modeling of the Synthesized Derivatives in Soybean LOX

All the synthesized derivatives have been studied for in silico docking. The molecular modeling study performed provided useful interpretation of the experimental results. The preferred docking orientation for the most potent derivative **3i** is shown in Figure 1. The binding of **3i** to soybean LOX (PDB code: 3PZW) has an AutoDock Vina score of −7.2. The docking scores of the novel derivatives are shown in Table 2. It is not possible to get an 100% relationship between in vitro lipoxygenase inhibitory activities and docking scores because the in vitro results represent experimental values while the docking scores are based on algorithms and scoring function calculations. Docking provides information about the binding value of the ligand with the protein. From the docking results it seems that the novel synthesized derivatives present allosteric interactions with the enzyme. Furthermore **3i** is able to accommodate the extensively hydrophobic cavity close to the active site with possible hydrophobic interactions (π-π stacking). Since most LOX inhibitors are antioxidants or free radical scavengers [40] lipoxygenation occurring via a carbon-centered radical on a lipid chain, it is likely that the extension scaffold of **3i** into the hydrophobic domain blocks approach of substrates to the active site and hence prevents oxidation by soybean LOX.

**Figure 2:**
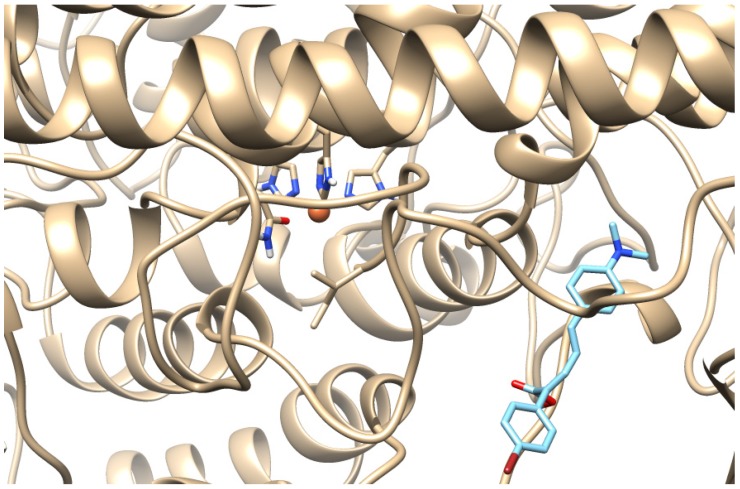
Docking pose of **3i** (depicted in blue) bound to soybean lipoxygenase (LOX-1). The iron ion is depicted as an orange sphere.

The molecular modeling study performed provided useful interpretation of the experimental results. The binding of **3i** to soybean LOX (PDB code: 3PZW) has a higher AutoDock Vina score than any of the other derivatives docked. The preferred docking orientation for the most potent derivative **3i** is shown in Figure 2.

## 3. Experimental Section

### 3.1. Materials and Instruments

All chemicals, solvents, chemical and biochemical reagents were of analytical grade and purchased from commercial sources (Merck, Merck KGaA, Darmstadt, Germany; Fluka, Sigma-Aldrich Laborchemikalien GmbH, Hannover, Germany; Alfa Aesar, Karlsruhe, Germany and Sigma-Aldrich, Louis, MO, USA). All starting materials were obtained from commercial sources and used without further purification. Soybean lipoxygenase, sodium linoleate, 2,2-azinobis-2-methyl-propanimidamine HCl (AAPH) were obtained from Sigma Chemical, Co. (St. Louis, MI, USA), while 1,1-diphenyl-2-picrylhydrazyl (DPPH), and nordihydroguairetic acid (NDGA) were purchased from the Aldrich Chemical Co. (Milwaukee, WI, USA).

Melting points (uncorrected) were determined on a MEL-Temp II (Lab Devices, Holliston, MA, USA). For the in vitro tests, UV-Vis spectra were obtained on a Perkin-Elmer 554 double beam spectrophotometer (Perkin-Elmer Corporation Ltd., Lane Beaconsfield, Bucks, UK). Infrared spectra (film as Nujol mulls or KBr pellets) were recorded with Perkin-Elmer 597 spectrophotometer (Perkin-Elmer Corporation Ltd., Lane Beaconsfield, Bucks, England).

^1^H-Nucleic Magnetic Resonance (^1^H-NMR) spectra were recorded at 300 MHz on a Bruker AMX300 spectrometer and at 500 MHz on a Bruker Avance 500 spectrometer (Bruker Analytische Messtechnik GmbH, Rheinstetten, Germany) in the stated solvent; chemical shifts are reported in δ (ppm) relative to the internal reference tetramethylsilane and coupling constants *J* in Hz. ^13^C-NMR spectra were obtained at 75.5 MHz (Bruker AM-300) and at 125 MHz (Bruker Avance 500 spectrometer) in CDCl_3_ solutions with tetramethylsilane as internal reference unless otherwise stated. Elemental analyses for C and H gave values acceptably close (±0.4%) to the theoretical values on a Perkin-Elmer 240B CHN analyzer. Reactions were monitored by thin layer chromatography on 5554 F_254_ Silica gel/TLC cards, Merck and Fluka). For preparative thin layer chromatography (PTLC) silica gel 60 F_254_, plates 2 mm, Merck KGa AICH078057 were used. For the experimental determination of the lipophilicity using reverse phase thin layer chromatography (RPTLC) 20 × 20 cm TLC-Silica gel 60 F_254_ DC Kieselgel plates (Merck) were used.

### 3.2. Chemistry General Procedures

Synthesis of 4-Bromo/hydroxyphenyl-substituted cinnamic acids **1i**–**7i** [17,18,19,28]. The title compounds were prepared by a Knoevenangel reaction, as shown in Scheme 1, according to literature methods [17,18,19]. A suitable aldehyde (0.015 mol) was condensed with 4-bromophenylacetic acid or 4-hydroxyphenylacetic acid (0.015 mol) and acetic acid anhydride (10 mL) in the presence of triethylamine (5 mL). The reaction mixture was refluxed for approximately 5 h (while monitoring completion of the reaction). The solution was poured into 2 N HCl, and ice and the precipitate formed was collected by filtration and recrystallized from 50% *v*/*v* aqueous ethanol. In case that no precipitate was formed an extraction with 3 × 100 mL CHCl_3_ was made and the organic phase was collected, dried over MgSO_4_ and evaporated to dryness affording a residue that was recrystallized from 50% aqueous ethanol.

*2-(4-Bromophenyl)-3-(3-phenoxyphenyl)acrylic acid* (**1i**). The crude product was recrystallized from 50% *v*/*v* aqueous ethanol. Yield: 59%; R_f_ (CH_3_COOCH_3_:petroleum ether:CHCl_3_, 2:3:1): 0.46; m.p.: 152–153 °C; ^1^H-NMR (500 MHz, CDCl_3_) δ 6.60 (1H, d, *J* = 10 Hz, aromatic proton), 6.85 (4H, m, aromatic protons), 6.99 (5H, m, aromatic protons), 7.41 (2H, m, aromatic protons), 7.62 (1H, s, CH=C), 7.86 (1H, m, aromatic proton); ^13^C-NMR (125 MHz) δ 119.1, 119.2, 119.3, 119.6, 122.5, 124.0, 125.6, 129.8, 129.9, 130.0, 130.1, 131.0, 131.9, 132.0, 133.8, 133.9, 135.6, 142.5, 156.9, 157.8, 172.1 (*C*OOH); Elemental Analysis: Expected (C_21_H_15_BrO_3_): C, 63.81; H, 3.83; Found % (C_21_H_15_BrO_3_): C, 63.87; H, 3.44.

*2-(4-Hydroxyphenyl)-3-(3-phenoxyphenyl)acrylic acid* (**1ii**). The crude product was extracted with 3 × 100 mL CHCl_3_ and the combined organic phase was collected, dried over MgSO_4_ and evaporated to dryness affording a residue that was recrystallized from 50% *v*/*v* aqueous ethanol. Yield: 33%; R_f_ (EtOH): 0.79; m.p.: 105–107 °C; ^1^H-NMR (500 MHz, CDCl_3_) δ 6.75 (2H, d, *J* = 8.4 Hz, aromatic protons), 6.86 (1H, d, *J* = 10 Hz, aromatic proton), 6.91 (2H, m, aromatic protons), 7.06 (2H, m, aromatic protons), 7.17 (3H, m, aromatic protons), 7.46 (3H, m, CH=C and aromatic proton), 7.85 (1H, d, *J* = 12 Hz, aromatic proton); ^13^C-NMR (125 MHz) δ 115.9, 119.2, 119.4, 119.5, 119.8, 120.1, 121.8, 123.7, 125.7, 129.7, 129.8, 129.87, 129.9, 130.9, 135.9, 141.6, 150.5, 156.3, 157.4, 157.6, 171.1 (*C*OOH); Elemental Analysis: Expected (C_21_H_16_O_4_): C, 75.89; H, 4.85; Found % (C_21_H_16_O_4_): C, 75.55; H, 4.45.

*3-(4-((4-Bromobenzyl)oxy)phenyl)-2-(4-bromophenyl)acrylic acid* (**2i**). The crude product was purified by PTLC. Yield: 22%; R_f_ (EtOAc:petroleum ether, 2:3): 0.70; ^1^H-NMR (500 MHz, CDCl_3_) δ 5.10 (2H, s, CH_2_), 7.15 (2H, m, aromatic protons), 7.19 (2H, m, aromatic protons), 7.30 (2H, m, aromatic protons), 7.43 (2H, m, aromatic protons), 7.52 (5H, m, CH=C, aromatic protons); ^13^C-NMR (125 MHz) δ 70.1 (CH_2_), 116.8, 122.7, 123.5, 126.0, 129.1, 129.9, 130.3, 130.4, 130.5, 130.6, 131.1, 131.4, 131.6, 131.8, 131.9, 132.1, 134.5, 135.7, 151.0, 162.0, 170.1 (*C*OOH); Elemental Analysis: Expected (C_22_H_16_Br_2_O_3_): C, 54.13; H, 3.30; Found % (C_22_H_16_Br_2_O_3_): C, 54.51; H, 3.70.

*3-(4-((4-Bromobenzyl)oxy)phenyl)-2-(4-hydroxyphenyl)acrylic acid* (**2ii**). The crude product was extracted with 3 × 100 mL CHCl_3_ and the combined organic phase was dried over MgSO_4_ and evaporated to dryness affording a residue that was recrystallized from 50% *v*/*v* aqueous ethanol. Yield: 7%; R_f_ (EtOAc:petroleum ether, 1:2): 0.78; ^1^H-NMR (300 MHz, CDCl_3_) δ 5.10 (2H, s, *C*H_2_), 6.75 (1H, d, *J* = 9 Hz, aromatic proton), 6.93 (1H, d, *J* = 9 Hz, aromatic protons), 7.1 (2H, m, aromatic protons), 7.3 (2H, m, aromatic protons), 7.5 (3H, m, CH=C, aromatic protons), 7.9 (4H, m, aromatic protons); ^13^C-NMR (75 MHz) δ 70.8 (*C*H_2_), 114.7, 115.0, 116.0, 120.3, 122.2, 127.0, 127.5, 129.3, 130.2, 131.1, 131.7, 132.8, 134.1, 134.6, 135.2, 137.2, 145.4, 150.6, 158.1, 160.1, 168.0 (*C*OOH); Elemental Analysis: Expected (C_22_H_17_BrO_4_): C, 62.13; H, 4.03; Found % (C_22_H_17_BrO_4_): C, 62.05; H, 3.91.

*2-(4-Bromophenyl)-5-(4-(dimethylamino)phenyl)penta-2,4-dienoic acid* (**3i**). The crude product was recrystallized from 50% *v*/*v* aqueous ethanol. Yield: 58%; R_f_ (EtOAc:petroleum ether, 1:5): 0.83; m.p.: 120–121 °C; ^1^H-NMR (500 MHz, CDCl_3_) δ 3.05 (6H, s, 2 × CH_3_), 6.77 (3H, m, CH=CH-CH, aromatic protons), 7.41 (8H, m, CH=CH-CH, aromatic protons); ^13^C-NMR (125 MHz) δ 39.8 (*C*H_3_), 40.0 (*C*H_3_), 115.9, 119.9, 121.5, 124.3, 130.1, 130.9, 131.1, 131.6, 132.1, 132.5, 133.1, 134.8, 136.5, 148.7, 168.3 (*C*OOH); Elemental Analysis: Expected (C_19_H_18_BrNO_2_): C, 61.30; H, 4.87; N, 3.76 Found % (C_19_H_18_BrNO_2_): C, 61.65; H, 4.48; N, 3.88.

*5-(4-(Dimethylamino)phenyl)-2-(4-hydroxyphenyl)penta-2,4-dienoic acid* (**3ii**). The crude product was recrystallized from 50% *v*/*v* aqueous ethanol. Yield: 64.2%; R_f_ (EtOAc:petroleum ether, 1:2): 0.81; m.p.: 204–205 °C; ^1^H-NMR (500 MHz, CDCl_3_) δ 3.20 (6H, s, 2 × CH_3_), 7.10 (5H, m, CH=CH-CH, aromatic protons), 7.71 (6H, m, CH=CH-CH, aromatic protons); ^13^C-NMR (125 MHz) δ 39.1 (*C*H_3_), 39.3 (*C*H_3_), 113.0, 114.0, 115.2, 115.3, 121.7, 127.2, 127.3, 129.2, 129.5, 131.4, 131.5, 133.0, 136.0, 138.0, 150.0, 156.0, 169.0 (*C*OOH); Elemental Analysis: Expected (C_19_H_19_NO_3_): C, 73.77; H, 6.19; N, 4.53; Found % (C_19_H_19_NO_3_): C, 73.61; H, 5.80; N, 4.22.

*4-Bromo-2-(4-bromophenyl)-5-phenylpenta-2,4-dienoic acid* (**4i**). The crude product was extracted with 3 × 100 mL CHCl_3_ and the combined organic phase was dried over MgSO_4_ and evaporated to dryness affording a residue that was recrystallized from 50% *v*/*v* aqueous ethanol. Yield: 98%; R_f_ (EtOAc: petroleum ether, 1:2): 0.88; m.p.: 211–212 °C; ^1^H-NMR (500 MHz, CDCl_3_) δ 6.10 (1H, s, PhCH=C(Br)=), 7.38 (3H, m, aromatic protons), 7.59 (3H, m, aromatic protons), 7.82 (4H, m, =C(Br)=CH, aromatic); ^13^C-NMR (125 MHz) δ 114.6, 124.0, 125.6, 127.7, 128.4, 128.6, 129.0, 129.1, 129.3, 129.4, 131.0, 131.7, 132.2, 133.2, 140.4 (Ph*C*H=C(Br)=), 147.1 (=C(Br)=*C*H), 168.6 (*C*OOH); Elemental Analysis: Expected (C_17_H_12_ Br_2_O_2_): C, 50.03; H, 2.96; Found % (C_17_H_12_ Br_2_O_2_): C, 50.28; H, 2.80.

*4-Bromo-2-(4-hydroxyphenyl)-5-phenylpenta-2,4-dienoic acid* (**4ii**). The crude product was extracted with 3 × 100 mL CHCl_3_ and the combined organic phase was dried over MgSO_4_ and evaporated to dryness affording a residue that was recrystallized from 50% *v*/*v* aqueous ethanol. Yield: 43%; R_f_ (EtOAc:petroleum ether, 2:3): 0.72; m.p.: 155–157 °C; ^1^H-NMR (500 MHz, CDCl_3_) δ 6.12 (1H, s, PhCH=C(Br)=), 6.79 (1H, d, *J =* 5 Hz, aromatic proton), 7.22 (4H, m, aromatic protons), 7.47 (5H, m, =C(Br)=CH, aromatic protons); ^13^C-NMR (125 MHz) δ 114.2, 116.0, 124.8, 125.6, 127.2, 127.9, 128.4, 129.0, 129.1, 129.3, 129.5, 130.8, 130.9, 133.3, 138.9 (Ph*C*H=C(Br)=), 140.5 (=C(Br)=*C*H), 168.8 (*C*OOH); Elemental Analysis: Expected (C_17_H_13_BrO_3_): C, 59.15; H, 3.80; Found % (C_17_H_13_BrO_3_): C, 59.38; H, 3.88.

*2-(4-Bromophenyl)-4-methyl-5-phenylpenta-2,4-dienoic acid* (**5i**). The crude product was extracted with 3 × 100 mL CHCl_3_ and the combined organic phase was dried over MgSO_4_ and evaporated to dryness affording a residue that was recrystallized from 50% *v*/*v* aqueous ethanol. Yield: 14%; R_f_ (EtOAc:petroleum ether, 1:5): 0.94; m.p.: 128–129 °C; ^1^H-NMR (500 MHz, CDCl_3_) δ 1.82 (3H, s, *C*H_3_), 6.52 (1H, s, PhCH=C(CH*3*)-CH=), 7.24 (8H, m, aromatic protons), 7.44 (2H, m, PhCH=C(CH_3_)-CH=, aromatic); ^13^C-NMR (125 MHz) δ 20.1 (*C*H_3_), 123.6, 127.7, 137.9, 128.4, 128.7, 130.1, 131.1, 131.3, 131.6, 131.8, 132.0, 134.3, 136.5, 136.6 (PhCH=*C*(CH_3_)-CH=), 138.0 (Ph*C*H=C(CH_3_)-*C*H=), 142.0 (PhCH=C(CH_3_)-CH=), 171.0 (*C*OOH); Elemental Analysis: Expected (C_18_H_15_BrO_2_): C, 62.99; H, 4.41; Found % (C_18_H_15_BrO_2_): C, 62.55; H, 3.97.

*2-(4-Hydroxyphenyl)-4-methyl-5-phenylpenta-2,4-dienoic acid* (**5ii**). The crude product was extracted with 3 × 100 mL CHCl_3_ and the combined organic phase was dried over MgSO_4_ and evaporated to dryness affording a residue that was recrystallized from 50% *v*/*v* aqueous ethanol. Yield: 8%; R_f_ (EtOAc:petroleum ether, 9:1): 0.83; m.p.: 90–91 °C; ^1^H-NMR (500 MHz, CDCl_3_) δ 1.58 (3H, s, *C*H_3_), 6.84 (3H, m, PhCH=C(CH*3*)-CH=, aromatic protons), 7.12 (3H, m, aromatic protons), 7.34 (4H, m, aromatic protons), 7.65 (1H, s, PhCH=C(CH_3_)-CH=); ^13^C-NMR (125 MHz) δ 21.2 (*C*H_3_), 115.6, 115.7, 122.0, 125.8, 127.6, 127.7, 127.9, 128.4, 128.5, 128.6, 128.7, 131.2, 131.5, 134.2 (PhCH=*C*(CH_3_)-CH=), 147.6 (Ph*C*H=C(CH_3_)-CH=), 157.4 (PhCH=C(CH_3_)-*C*H=), 169.3 (*C*OOH); Elemental Analysis: Expected (C_18_H_16_O_3_): C, 77.12; H, 5.75; Found % (C_18_H_15_BrO_2_): C, 76.96; H, 5.36.

*2-(4-Bromophenyl)-3-(3,5-di-tert-butyl-2-hydroxyphenyl)acrylic acid* (**6i**). The crude product was recrystallized from 50% *v*/*v* aqueous ethanol. Yield: 87%; R_f_ (EtOAc:petroleum ether, 1:4): 0.84; m.p.: 168–170 °C; ^1^H-NMR (500 MHz, CDCl_3_) δ 1.35 (18H, s, 6 × CH_3_), 7.11 (2H, m, aromatic protons), 7.41 (3H, m, aromatic protons), 7.49 (2H, m, Z-CH=, aromatic); ^13^C-NMR (125 MHz) δ 31.4 (3 × *C*H_3_), 35.5 (3 × *C*H_3_), 122.2, 124.6, 124.7,127.1, 128.0, 129.8, 130.8, 130.9, 131.8, 131.9, 132.2, 132.4, 134.9, 142.9, 144.0, 149.6, 171.5 (*C*OOH); Elemental Analysis: Expected (C_23_H_27_BrO_3_): C, 64.04; H, 6.31; Found % (C_18_H_15_BrO_2_): C, 64.15; H, 5.94.

*3-(3,5-di-tert-Butyl-2-hydroxyphenyl)-2-(4-hydroxyphenyl)acrylic acid* (**6ii**). The crude product was recrystallized from 50% *v*/*v* aqueous ethanol. Yield: 53%; R_f_ (EtOAc:petroleum ether, 1:4): 0.75; m.p.: 134–136 °C; ^1^H-NMR (500 MHz, CDCl_3_) δ 1.38 (18H, brs, 6 × CH_3_), 7.00 (2H, m, aromatic protons), 7.13 (2H, br, aromatic protons), 7.6 (2H, m, aromatic protons), 7.6 (1H, s, Z-CH=); ^13^C-NMR (125 MHz) δ 31.4 (3 × *C*H_3_), 35.5 (3 × *C*H_3_) 121.8, 122.2, 124.6, 124.7, 127.3, 129.7, 130.7, 130.8, 131.1, 131.2, 134.2, 142.8, 142.9, 150.6, 150.7, 169.5, 171.2 (*C*OOH); Elemental Analysis: Expected (C_23_H_28_O_4_): C, 74.97; H, 7.66; Found % (C_23_H_28_O_4_): C, 74.59; H, 7.33.

*2-(4-Bromophenyl)-3-(3,5-di-tert-butyl-4-hydroxyphenyl)acrylic acid* (**7i**). The crude product was recrystallized from 50% *v*/*v* aqueous ethanol. Yield: 76%; R_f_ (EtOAc:petroleum ether, 3:1): 0.90; m.p.: 137–138 °C; ^1^H-NMR (500 MHz, CDCl_3_) δ 1.34 (9H, s, 3 × C*H_3_*), 1.51 (9H, s, 3 × C*H_3_*), 7.08 (2H, m, aromatic protons), 7.43 (2H, m, aromatic protons), 7.57 (2H, m), 7.80 (1H, s Z-CH=); ^13^C-NMR (125 MHz, CDCl_3_) δ 31.5 (3 × *C*H_3_), 35.2 (3 × *C*H_3_), 122.9, 124.3, 125.9, 129.9, 130.1, 130.3, 131.3, 132.5, 133.9, 137.2, 141.4, 146.9, 150.1, 160.2 (*C*OOH); Elemental Analysis: Expected (C_23_H_27_BrO_3_): C, 64.04; H, 6.31; Found % (C_23_H_27_BrO_3_): C, 64.15; H, 5.94.

*3-(3,5-di-tert-Butyl-4-hydroxyphenyl)-2-(4-hydroxyphenyl)acrylic acid* (**7ii**). The crude product was recrystallized from 50% *v*/*v* aqueous ethanol. Yield: 39%; R_f_ (EtOAc:petroleum ether, 3:1): 0.88; m.p.: 139–140 °C; ^1^H-NMR (500 MHz, CDCl_3_) δ 1.37 (9H, s, 3 × C*H_3_*), 1.55 (9H, s, 3 × C*H_3_*), 7.18 (2H, m, aromatic protons), 7.35 (2H, m, aromatic protons), 7.54 (1H, s, aromatic), 7.74 (2H, m, Z-CH= and aromatic); ^13^C-NMR (125 MHz) δ 30.0, 31.5, 34.8, 119.5, 121.7, 122.6, 126.2, 126.7, 132.7, 137.1, 141.4, 146.9, 150.4, 151.0, 160.3, 169.5; Elemental Analysis: Expected (C_23_H_28_O_4_): C, 74.97; H, 7.66; Found % (C_23_H_28_O_4_): C, 74.60; H, 7.59.

### 3.3. Physicochemical Studies

#### 3.3.1. Determination of R_M_ Values

Reversed phase TLC (RPTLC) was performed on silica gel plates impregnated with 55% (*v*/*v*) liquid paraffin in light petroleum ether. The mobile phase was a methanol/water mixture (77/23, *v*/*v*) containing 0.1% of acetic acid. The plates were developed in closed chromatography tanks saturated with the mobile phase at 24 °C. Spots were detected under UV light. R_M_ values were determined from the corresponding R_f_ values (from five individual measurements) using the equation R_M_ = log[(1/R_f_) − 1] [17,18,28].

#### 3.3.2. In Silico Determination of Lipophilicity as Clog *P*

The CLOGP Program of Biobyte Corp was used for the theoretical calculation of lipophilicity as Clog *P* values [41].

### 3.4. Biological In Vitro Assays

The in vitro assays were performed at a concentration of 100 µM (a stock solution 10 mM in DMSO was used) at least in triplicate and the standard deviation of absorbance was less than 10% of the mean. The compounds were diluted as 0.1% DMSO under sonification in the appropriate buffer in several dilutions.

#### 3.4.1. Determination of the Reducing Activity of the Stable Radical 1,1-Diphenyl-picrylhydrazyl (DPPH)

To a solution of DPPH in absolute ethanol an equal volume of the compounds dissolved in DMSO was added. The final concentrations of the measured solutions of the novel acids were 50 and 100 µM starting from a stock solution (10 mM) in DMSO. The absorbance was recorded at 517 nm, after 20 and 60 min at room temperature (Table 1). NDGA was used as reference compound [17,18,21,28].

#### 3.4.2. Competition of the Tested Compounds with DMSO for Hydroxyl Radicals

The hydroxyl radicals generated by the Fe^3+^/ascorbic acid system, were detected by the determination of formaldehyde produced from the oxidation of DMSO (Table 2). Trolox was used as reference compound [17,18,28].

#### 3.4.3. Non Enzymatic Assay of Superoxide Radical Scavenging Activity

The superoxide radicals are generated by mixing *N*-methylphenazonium methyl sulfate (PMS), NADH and air–oxygen and determined by the nitroblue tetrazolium method and measured at 560 nm (Table 2). Caffeic acid was used as reference compound [17,18,28].

#### 3.4.4. ABTS^+•^—Decolorization Assay for Antioxidant Activity

In order to produce the ABTS radical cation (ABTS^+•^), ABTS stock solution in water (7 mM) was mixed with potassium persulfate (2.45 mM) and left in the dark at room temperature for 12–16 h before use. Our published experimental technique was used (Table 2). The results are recorded after 1 min of the mixing solutions at 734 nm. The results were compared to the appropriate standard inhibitor Trolox [28].

#### 3.4.5. Inhibition of Linoleic Acid Peroxidation

2,2’-Azobis(2-amidinopropane) dihydrochloride (AAPH) was used as a free radical initiator to start the oxidation reaction. The method was based on the production of conjugated diene hydroperoxide by oxidation of linoleic acid at 234 nm expressed as an increased absorption. Trolox was used as reference compound (93%) (Table 2) [28].

#### 3.4.6. Soybean Lipoxygenase Inhibition Study In Vitro

In vitro study was evaluated as reported previously by our group [17,18,28]. The tested compounds were incubated at room temperature with sodium linoleate (0.1 mM) and 0.2 mL of enzyme solution (1/9 × 104 *w*/*v* in saline). The method was based to the conversion of sodium linoleate to 13-hydroperoxylinoleic acid at 234 nm. NDGA (IC_50_ = 0.45 μM) a standard inhibitor was used as reference (Table 1). In order to determine the IC_50_ values different concentrations were used. The results are given in Table 1.

### 3.5. Computational Methods—Molecular Docking Studies on Soybean Lipoxygenase

UCSF Chimera was used for the visualization of the protein (PDB code: 3PZW, University of California, San Francisco, CA, USA) [42]. Water molecules were removed, missing residues were added with Modeller [43], hydrogen atoms and AMBER99SB-ILDN charges were added, and the charge on iron was set to +2.0, with no restraint applied to the iron atom and the ligands. Open Babel (OpenEye Scientific, Santa Fe, NM, USA) was used to generate and minimize ligand 3D coordinates using the MMFF94 force field [44]. Ligand topologies and parameters were generated by ACPYPE (AnteChamber PYthon Parser interface, GNU GPL v3, https://www.researchgate.net/publication/229552001_ACPYPE-Antechamber_python_parser_interface) [45] using Antechamber [46]. Energy minimizations were carried out using the AMBER99SB-ILDN force field [47] with GROMACS 4.6, Biophysical Chemistry department of University of Groningen, Municipality, The Netherlands. Docking was performed with AutoDock Vina (1.1.2, Molecular Graphics Lab at the Scripps Research Institute, San Diego, CA, USA) [48] applying a grid box of size 100 Å, 70 Å, 70 Å in X, Y, Z dimensions. The generation of docking input files and the analysis of the docking results was accomplished by UCSF-Chimera. Docking was carried out with an exhaustiveness value of 10 and a maximum output of 20 docking modes.

## 4. Conclusions

The novel designed and synthesized derivatives present multitarget activity against several biological targets implicated in inflammation and allergy, e.g., lipoxygenase, anti-lipid peroxidation and strong antioxidant capacity. The compounds presented medium antioxidant activity using DPPH. Small differences with time and concentration were observed. All the synthesized derivatives strongly scavenged hydroxyl radicals at 100 μM while they reduced moderately at 100 μΜ the ABTS radical cation (ABTS^+•^). The majority of the synthesised derivatives inhibited lipid peroxidation in a concentration-dependent manner. Compound **3i** seems to be the most potent lipoxygenase inhibitor with IC_50_ = 7.4 µM. The antioxidant results support the LOX inhibitory activities. Docking studies of the most potent derivative **3i** proved that allosteric interactions govern the LOX inhibitory binding activity.

## Figures and Tables

**Table 1:** In vitro lipoxygenase (LOX) inhibitory activity at 0.01 µM or IC_50_ (µM). % Interaction with the stable radical 1,1-diphenyl-picrylhydrazyl (DPPH). Lipophilicity values: experimental R_M_ and determination of lipophilicity as clog *P*.

Compd.	Clog *P* ^a^	R_M_^b^ (±SD) ^c^	LOX ^d,e^ IC_50_ (μM) (% Inhibition 100 µM)	RA% 50 µM 20 min	RA% 50 µM 60 min	RA% 100 µM 20 min	RA% 100 µM 60 min
**1i**	6.25	−0.103 (0.009)	100	38	37	44	46
**1ii**	4.72	−0.537 (0.002)	91.5	40	39	45	47
**2i**	7.00	0.605 (0.016)	76.0	35	34	37	40
**2ii**	5.47	0.061 (0.007)	77.0	36	37	43	56
**3i**	5.07	0.582 (0.037)	7.4	34	37	48	51
**3ii**	3.54	−0.642 (0.032)	89.0	43	35	44	46
**4i**	5.40	0.423 (0.017)	28%	30	30	38	36
**4ii**	3.87	0.011 (0.001)	69.0	34	46	35	37
**5i**	5.30	0.683 (0.015)	77.5	33	33	36	38
**5ii**	3.77	−0.565 (0.003)	81.5	32	39	30	41
**6i**	7.33	0.480 (0.035)	60.0	38	36	44	39
**6ii**	5.80	0.622 (0.025)	74.0	43	36	31	36
**7i**	7.24	1.151 (0.021)	72.5	34	35	30	31
**7ii**	5.71	0.654 (0.016)	57.5	33	35	33	34
**NDGA**	-	-	0.45	81	83	87	93

^a^ Theoretically calculated clog *P* values; ^b^ R_M_ values are the average of at least 5 measurements; ^c^ SD standard deviation <10%; ^d^ Soybean lipoxygenase inhibition expressed as IC_50_ (μM). IC_50_ represents the concentration of the tested compounds that is required for 50% inhibition in vitro. ^e^ Values are means (±SD < 10%) of three or four different determinations, (*p* = 0.05).

**Table 2:** In vitro % radical scavenging activity, hydroxyl (HO^•^%) and superoxide (O_2_^−^^•^ %) radicals, % Decolorization activity ABTS^+•^%, % anti-lipid peroxidation (AAPH) assays, docking scores.

Compd.	HO^•^% ^a^ 100 µM	O_2_^−•^% ^a^ 100 µM	ABTS^+•^% ^a^ 100 µM	AAPH% ^a^ 100 µM	AAPH% ^a^ 50 µM	Docking Scores
**1i**	94	54	29	90	34	−9.5
**1ii**	100	8	83	94	22	−8.0
**2i**	95	na	32	96	48	−9.5
**2ii**	99	na	47	87	49	−9.2
**3i**	100	na	83	88	43	−7.2
**3ii**	99	85	63	95	71	−7.4
**4i**	83	46	24	85	52	−6.6
**4ii**	96	100	24	97	59	−8.6
**5i**	82	na	30	98	10	−7.3
**5ii**	89	8	35	93	53	−7.0
**6i**	100	na	53	100	56	−7.2
**6ii**	100	na	38	96	64	−6.9
**7i**	87	54	28	100	61	−7.2
**7ii**	72	69	34	100	69	−9.3
**Trolox**	73	-	88	93	-	-
**CA**		23				

na: no activity under the used experimental conditions; ^a^ Values are means (±SD < 10%) of three or four different determinations, (*p* = 0.05).
